# Depression does not predict clinical outcome of Chinese peritoneal Dialysis patients after adjusting for the degree of frailty

**DOI:** 10.1186/s12882-020-01994-4

**Published:** 2020-08-05

**Authors:** Gordon Chun-Kau Chan, Jack Kit-Chung Ng, Kai-Ming Chow, Bonnie Ching-Ha Kwan, Vickie Wai-Ki Kwong, Wing-Fai Pang, Phyllis Mei-Shan Cheng, Man-Ching Law, Chi-Bon Leung, Philip Kam-Tao Li, Cheuk-Chun Szeto

**Affiliations:** Carol & Richard Yu Peritoneal Dialysis Research Centre, Department of Medicine & Therapeutics, The Chinese University of Hong Kong, Hongkong, Shatin China

## Abstract

**Background:**

Depression and frailty contribute to the adverse clinical outcome of peritoneal dialysis (PD) patients. However, the interaction between depression and frailty in PD patients remains uncertain. We determined the prevalence of depression and frailty in prevalent Chinese PD patients, dissected the internal relationship between depression and frailty, and determined their relative contribution to the adverse clinical outcome in PD patients.

**Methods:**

In a prospective observational study, we recruited 267 prevalent PD patients. Depression was identified by Patient Health Questionnaire (PHQ-9). Frailty was identified by a validated Frailty Score. All cases were followed for one year. Outcome measures included number and duration of hospitalization, peritonitis rate, and all-cause mortality.

**Results:**

Of the 267 patients, 197 patients (73.8%) were depressed, and 157 (58.8%) were frail. There was a substantial overlap between depression and frailty. Although depression and frailty were associated the number and duration of hospitalization by univariate analysis, the association became insignificant after adjusting for confounding factors by multivariate analysis. Both depression and frailty were associated with one-year mortality by univariate analysis. One-year patient survival was 95.9, 86.5, 82.4 and 71.0% for patients with nil, mild, moderate and severe frailty, respectively (*p* = 0.001). Frailty was an independent predictor of patient survival by multivariate analysis (adjusted hazard ratio 1.424, 95% confidence interval 1.011–2.005. *p* = 0.043), while the prognostic effect of depression disappears after adjusting for frailty score.

**Conclusion:**

Depression and frailty were common among Chinese PD patients. Frailty, but not depression, was an independent predictor of one-year mortality.

## Background

Depression and frailty are common problems in the general population and dialysis patients. The worldwide prevalence of depression in dialysis patients was 22.8% [1]. In a study conducted in Hong Kong, the prevalence of major depressive episode among peritoneal dialysis (PD) population was 16% [2]. Depressed PD patients usually suffer from multiple physical and psychological symptoms, all of which affect their physical function, quality of life, and impede on their adherence to dialysis regimen [3]. A study in Hong Kong showed that 55% of PD patients were classified as having high emotional distress, which was an independent risk factor for the development of peritonitis [4]. Another meta-analysis also showed that the presence of depressive symptoms was a significant predictor of mortality in dialysis patients [5].

In addition to depression, it is increasingly recognized that physical frailty is an important contributing factor to the adverse clinical outcome of dialysis patients [6–8]. Frailty was present in 15% of patients with mild to moderate chronic renal insufficiency [9], and 67.7% of dialysis patients [10]. A large cohort study in United States reported that frailty was associated with female sex, older age, and a higher comorbidity burden [11]. For dialysis patients, prior studies showed that patients who were on hemodialysis were more likely to be frail than those who were on PD [10]. Our previous study showed that frail PD patients have a high risk of requiring hospitalization and their hospital stay tends to be prolonged [6].

Although both depression and frailty are common, their relation in dialysis patients have not been well studied. Iyasere et al. [12] showed that patients on assisted PD had a higher prevalence of possible depression and higher Hospital Anxiety and Depression Scale (HADS) depression score than in-center hemodialysis patients, but their frailty scores were similar. Our previous study showed that around 40% frail PD patients had depressive symptoms, while depression was rare in PD patients without frailty [13]. More importantly, physical frailty and depressive symptoms had additive adverse effect on the nutritional status and clinical outcome [13]. However, our previous study used the Geriatric Depression Scale (GDS) for the screening of depression [14], which may not be appropriate for middle-age dialysis patients. Moreover, frailty and depression were both defined as dichotomized entities in that study [13], so that the severity of either problem could not be considered. In the present study, we determined correlation between depression and frailty in prevalent Chinese PD patients, and explored their interaction on the peritonitis rate, hospitalization, and short-term mortality.

## Methods

### Study population

We recruited 267 prevalent PD patients from a single dialysis unit between 2015 and 2016. Patients with expected survival of less than 3 months, or those who were planned to receive kidney transplantation in 3 months, were excluded. The study was approved by the Joint Chinese University of Hong Kong - New Territories East Cluster Clinical Research Ethics Committee. All study procedures were in compliance with the Declaration of Helsinki.

### Data collection

After written informed consent, clinical and laboratory data were obtained by chart review. Clinical data, including patient’s age, gender, body weight, height, primary diagnosis of renal disease, concomitant chronic medical illnesses including diabetes mellitus, ischemic heart disease, cerebrovascular accident, peripheral vascular disease, chronic hepatitis B and C infection, chronic lung disease, malignancy and immunological diseases, were recorded by chart review. Laboratory data, including serum albumin, lipid profile, total weekly Kt/V, residual glomerular filtration rate, were retrieved from our computerized clinical management system. The Charlson Comorbidity Index (CCI) was computed to represent the comorbidity load [15]. For nutritional assessment, we used the Comprehensive Malnutrition Inflammation Score (MIS) [16] and Subjective Global Assessment (SGA) [17].

### Assessment of depression

We used the Patient Health Questionnaire (PHQ-9) for screening and classification of depression [18]. PHQ-9 consists of 9 questions corresponding to the 9 criteria for defining depression according to Diagnostic and Statistical Manual Fourth Edition (DSM-IV). Each question was scored from 0 point (i.e. not at all) to 3 points (i.e. nearly every day) according to severity. Overall score was computed and patients were classified according to their severity of depressive symptoms, from none, mild, moderate, moderately severe, and severe (with PHQ-9 score of 0 to 4, 5 to 9, 10 to 14, 15 to 19, and ≥ 20, respectively) [18].

### Assessment of frailty

We used a previously published Chinese questionnaire that consisted of 30 yes/no questions (Supplementary File 1) [6, 13]. The questions involve subjective assessment of personal health, psychological state, physical state in terms of the patients’ capacity to perform various daily activities. Patients were classified accordint to their degree of frailty, with a score 5 or below as nil, score 6 to 8 as mild, score 9 to 11 as moderate, and score 12 or above as severe.

### Outcome measures

After the baseline assessment, all patients were followed for 1 year. The primary outcomes of this study included the number of hospitalization and total duration of hospitalization for all causes, the number of peritonitis episodes, and all-cause mortality. Peritonitis was diagnosed according to the standard guideline [19]. The peritonitis rate was represented as the number of peritonitis episode per patient-year of follow up. Technique survival was added post hoc as a secondary outcome measure, and technique failure was defined as transfer to long-term hemodialysis. Censoring events for technique survival include kidney transplant, recovery of renal function, loss to follow up, and transfer to other dialysis centers.

### Statistical analysis

Statistical analysis was performed by SPSS for Windows software version 24 (SPSS Inc., Chicago). All study data are listed in Supplementary File 2. Descriptive data were presented as mean ± SD if normally distributed, or median (inter-quartile range) otherwise. Patients were grouped according to the degree of depression and frailty as defined above for analysis. Baseline clinical parameters between depression and frailty groups were compared by Kendall’s tau test and Spearman rank order correlation coefficient as appropriate, with post hoc subgroup analysis, when needed, by Student’s t-test or one way analysis of variance (ANOVA) for continuous variables, and Chi-square test for categorical variables.

The number of hospital admission and duration of hospitalization were compared between frailty groups by Spearman’s rank correlation. To adjust for clinical confounding factors, the log-linear regression model was then used to because the data were highly skewed. Potential confounders, including age, duration of dialysis, body weight, Charlson’s comorbidity score, serum albumin, total Kt/V, and residual GFR, were added to the model. Backward stepwise elimination was used to determine the independent predictor of hospitalization.

Kaplan-Meier method was used to present the data of patient and technique survival, and log-rank test was used to compare between survival curves. The Cox proportional hazards model was then used to adjust for potential confounders and identify independent predictors of patient survival. In addition to the degree of frailty and depression being added separately, the Cox models were constructed by similar clinical parameters used in the analysis of hospitalization. These parameters were selected because of their reported significance in determining the prognosis of PD patients. Backward stepwise elimination was applied to remove insignificant variables. *P* < 0.05 was considered to be statistically significant. All probabilities were two-tailed.

## Results

A total of 267 patients were recruited. Their baseline clinical and demographic data are summarized in Table 1. According to the PHQ-9 score, 110 patients (41.2%) were classified as not depressed, 80 (30.0%) were mildly depressed, 43 (16.1%) were moderately depressed, 26 (9.7%) were moderate-to-severely depressed, and 8 (3.0%) were severely depressed. The demographic data, clinical and biochemical parameters are compared between groups with different degree of depression and summarized in Table 1.

**Table 1:** Baseline clinical and demographic data according to severity of depression

	All patients	Depression					*P* value
		none	mild	moderate	moderately severe	severe	
No. of patients	267	110	80	43	26	8	
Age (year)	62.9 ± 12	62.3 ± 12.7	62.2 ± 11.6	63.5 ± 12.1	64.0 ± 9.7	70.1 ± 12.5	*p* = 0.3^b^
Sex (M:F)	131:136	57:53	36:44	25:18	11:15	2:6	p = 0.5^a^
Weight (kg)	64.9 ± 13.1	65.4 ± 13.7	63.3 ± 11.6	66.2 ± 15.0	66.1 ± 12.2	61.9 ± 14.3	p = 0.9^b^
Height (cm)	160.2 ± 7.8	161.0 ± 7.8	159.0 ± 7.5	162.0 ± 7.5	159.0 ± 8.9	158.0 ± 6.4	*p* = 0.4^b^
Duration of dialysis (months)	30.9 (13.8–62.1)	29.6 (14.9–55.7)	30.5 `(13.1–62.5)	42.7 (17.1–70.5)	22.4 (12.9–59.7)	38.4 (35.4–49.3)	p = 0.8^b^
Renal diagnosis, no. of patient (%)							*p* = 0.17^a^
diabetic nephropathy	98 (36.7%)	37 (33.6%)	27 (33.8%)	20 (46.5%)	9 (34.6%)	5 (62.5%)	
hypertensive nephrosclerosis	30 (11.2%)	11 (10%)	9 (11.3%)	6 (14%)	4 (15.4%)	0 (0%)	
glomerulonephritis	84 (31.5%)	38 (34.5%)	25 (31.3%)	10 (23.3%)	8 (30.8%)	3 (37.5%)	
polycystic kidney	8 (3.0%)	4 (3.6%)	3 (3.8%)	1 (2.3%)	0 (0%)	0 (0%)	
urological problems	11 (4.1%)	9 (8.2%)	0 (0%)	1 (2.3%)	1 (3.8%)	0 (0%)	
unknown	30 (11.2%)	10 (9.1%)	14 (17.5%)	4 (9.3%)	2 (7.7%)	0 (0%)	
others	6 (2.2%)	1 (0.9%)	2 (2.5%)	1 (2.3%)	2 (7.7%)	0 (0%)	
Comorbidity, no. of case (%)							
diabetes	121 (45.3%)	47 (42.7%)	34 (42.5%)	23 (53.5%)	11 (42.3%)	6 (75%)	p = 0.3^a^
ischemic heart disease	29 (10.9%)	16 (14.5%)	13 (16.3%)	6 (14.0%)	3 (11.5%)	0 (0%)	p = 0.5^a^
cerebrovascular disease	47 (17.6%)	16 (14.5%)	15 (18.8%)	11 (25.6%)	3 (11.5%)	2 (25%)	p = 0.3^a^
peripheral vascular disease	9 (3.4%)	4 (3.6%)	0 (0%)	1 (2.3%)	3 (11.5%)	1 (12.5%)	p = 0.4^a^
Charlson Comorbidity Index	5.66 ± 2.51)	5.45 ± 2.26	5.25 ± 2.32	5.37 ± 2.12	4.81 ± 1.72	6.50 ± 2.56	p = 0.4^b^
PD exchange by assistants, no. of case (%)	34 (12.7%)	10 (9.1%)	9 (11.3%)	8 (18.6%)	2 (7.7%)	5 (37.5%)	*p* = 0.14^a^
Baseline biochemistry							
serum albumin (g/L)	33.7 ± 4.37	34.1 ± 4.57	34.2 ± 3.92	31.8 ± 4.39	35.0 ± 3.15	28.4 ± 3.69	*p* = 0.09^b^
total cholesterol (mmol/l)	4.91 ± 1.35	4.95 ± 1.28	4.77 ± 1.34	4.86 ± 1.54	5.13 ± 1.39	5.08 ± 1.44	*p* = 0.9^b^
LDL cholesterol (mmol/l)	2.82 ± 1.13	2.82 ± 1.04	2.66 ± 1.09	2.92 ± 1.29	3.06 ± 1.20	2.89 ± 1.43	p = 0.8^b^
HDL cholesterol (mmol/l)	1.31 ± 0.57	1.32 ± 0.46	1.28 ± 0.53	1.21 ± 0.49	1.36 ± 0.48	1.87 ± 1.70	*p* = 0.5^b^
weekly Kt/V	1.82 ± 0.48	1.89 ± 0.53	1.81 ± 0.49	1.58 ± 0.36	1.86 ± 0.37	1.86 ± 0.47	*p* = 0.08^b^
residual GFR (ml/min/1.73m^2^)	1.46 ± 2.00	1.87 ± 2.25	1.34 ± 1.73	0.85 ± 1.87	1.33 ± 1.67	0.39 ± 0.81	p = 0.003^b^

*LDL* low density lipoprotein, *HDL* high density lipoprotein. Data are expressed as mean ± standard deviation or median (inter-quartile range), and compared by ^a^Kendall’s tau test and ^b^Spearman rank order correlation coefficient

As for the severity of frailty according to the Frailty Score (FQ), 73 patients (27.3%) were classified as not frail, 74 (27.7%) mildly frail, 51 (19.1%) moderately frail, and 69 (25.8%) severely frail. There was a significant correlation between the Frailty Score and Charlson comorbidity score (r = 0.674, *p* < 0.0001). Further collinearity test confirmed absence of multicollinearity between frailty and Charlson Comorbidity Index (Collinearity statistics, VIF 1.619). A history of cerebrovascular disease was associated with the severity of frailty (*p* < 0.001).

**Fig. 1:**
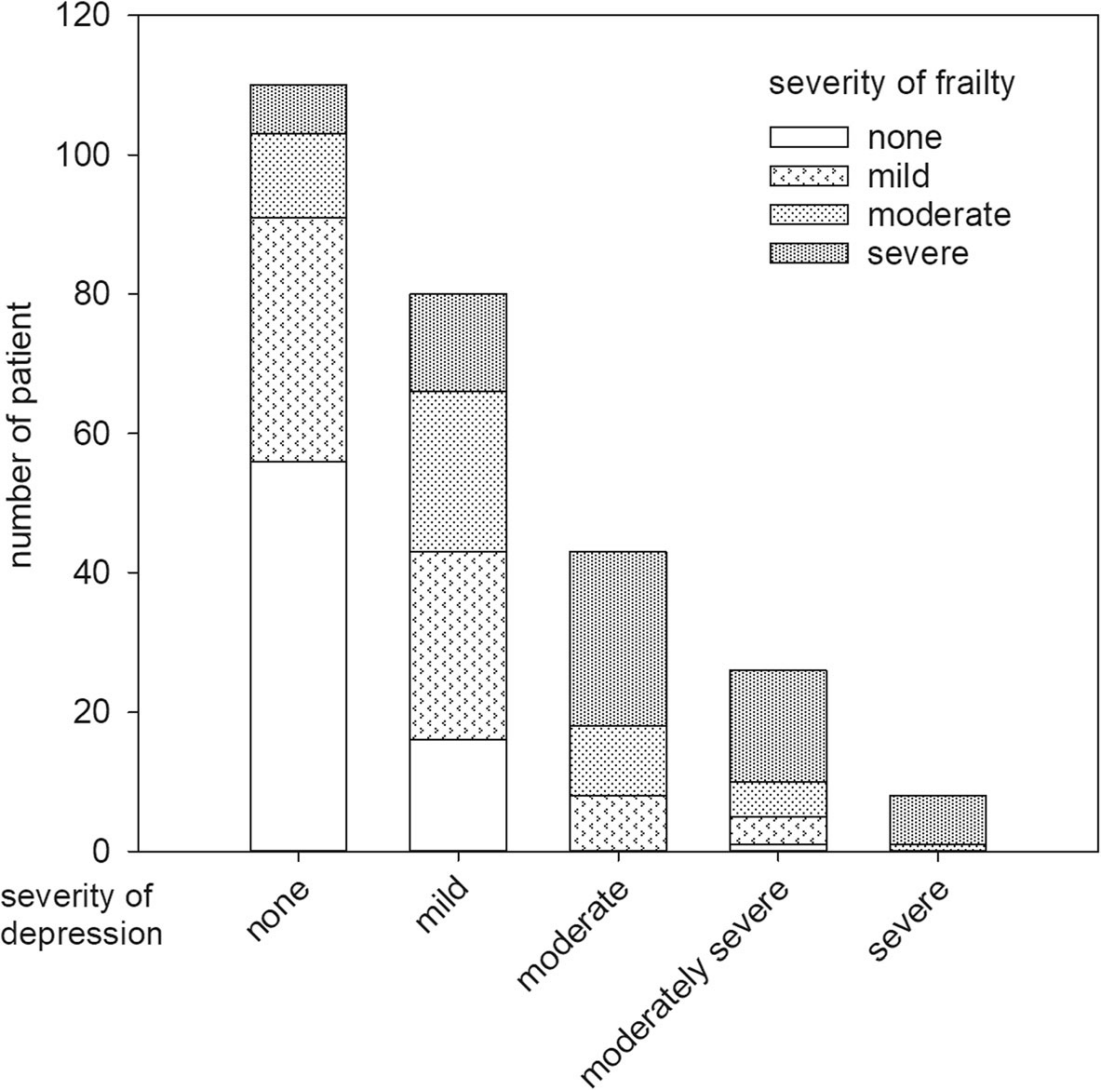
Overlap between depression and frailty. There is a substantial overlap between depression and frailty as shown in the diagram. The percentage of frailer cases increased with severity of depression in a stepwise manner

There was a substantial overlap between depression and frailty (Fig. 1). In essence, 157 patients (58.8%) with PHQ-9 score > 4 were classified as depressed, whereas 194 patients (72.7%) with frailty score > 5 and were classified as frail; 140 patients (52.4%) are both depressed and frail. The proportion of cases with higher frailty score increases with severity of depression. There was a significant correlation between PHQ-9 score and frailty score (Spearman’s r = 0.661, *p* < 0.0001).

### Hospitalization and peritonitis

After 1 year of follow-up, there were 749 hospital admissions for a total of 5224 days; 72 patients (27.0%) did not require hospitalization. The relation between hospitalization and depression and frailty are shown in Tables 2 and 3, respectively. In short, the PHQ-9 score had a modest but statistically significant correlation with the number of hospitalization (r = 0.164, *p* = 0.007) and duration of hospitalization (r = 0.147, *p* = 0.016). Similarly, the frailty score also had a modest but significant correlation with the number of hospitalization (r = 0.220, *p* < 0.0001), and duration of hospitalization (r = 0.176, *p* = 0.004). However, after adjusting for potential confounding factors by multivariate log linear regression analysis, neither depression nor frailty remained as an independent predictor for number of hospitalization or duration of hospitalization. In this model, only Charlson comorbidity index, body weight, and residual GFR had significant independent association with the number of hospital admission (unstandardized B: Charlson Comorbidity Index, 0.052, 95% confidence interval [CI] 0.012–0.092, *p* = 0.012; body weight, 0.008. 95% CI 0.001–0.016, *p* = 0.037; residual GFR, − 0.079, 95% CI -0.129 - -0.028, *p* = 0.002) as well as the duration of hospitalization (unstandardized B: Charlson Comorbidity Index, 0.095, 95% CI 0.016–0.173, *p* = 0.018; body weight, 0.020, 95% CI 0.005–0.035, *p* = 0.011; residual GFR, − 0.130, 95% CI -0.231 – 0.029, p = 0.012).

**Table 2:** Relation between depression, hospitalization, and peritonitis rate

Depression	none	mild	moderate	moderately severe	severe	*P*-value
No of patients	110	80	43	26	8	
Without hospitalization, no. of patients (%)	35 (31.8%)	21 (26.3%)	10 (23.3%)	6 (23.1%)	0	*p* = 0.075^a^
Number of hospitalization	2.29 ± 2.67	2.84 ± 3.20	3.30 ± 3.48	3.35 ± 3.22	5.13 ± 3.87	p = 0.007^b^
Duration of hospitalization (days)	6.0 (0.0–21.5)	6.5 (0.8–23.0)	9.0 (1.0–26.0)	16.5 (3.0–30.5)	47.0 (21.5–88.3)	p = 0.021^b^
Peritonitis-free, no. of patients (%)	84 (76.4%)	60 (75.0%)	33 (76.7%)	18 (69.2%)	8 (100%)	p = 0.9^a^
Number of peritonitis episode (per year)	0.36 ± 0.77	0.35 ± 0.66	0.30 ± 0.60	0.42 ± 0.70	0	p = 0.9^b^

Data are expressed as mean ± standard deviation or median (inter-quartile range), and compared by ^a^Kendall’s tau test and ^b^Spearman rank order correlation coefficient

**Table 3:** Relation between frailty, hospitalization, and peritonitis rate

Frailty	none	mild	moderate	severe	*P*-value
No of patients	73	74	51	69	
Without hospitalization, no. of patients (%)	22 (30.1%)	24 (32.4%)	15 (29.4%)	11 (15.9%)	*p* = 0.058^a^
Number of hospitalization	2.10 ± 2.54	2.26 ± 2.90	3.35 ± 3.29	3.74 ± 3.43	p < 0.0001^b^
Duration of hospitalization (days)	7.0 (0.0–15.0)	5.0 (0.0–17.0)	11.0 (0.0–30.5)	14.0 (3.0–32.0)	p = 0.004^b^
Peritonitis-free, no. of patients (%)	56 (76.7%)	60 (81.1%)	34 (66.7%)	53 (76.8%)	*p* = 0.6^a^
Number of peritonitis episode (per year)	0.27 ± 0.53	0.32 ± 0.85	0.45 ± 0.70	0.33 ± 0.66	p = 0.5^b^

Data are expressed as mean ± standard deviation or median (inter-quartile range), and compared by ^a^Kendall’s tau test and ^b^Spearman rank order correlation coefficient

During the study period, there were 90 peritonitis episodes; 203 patients (76.0%) were free of peritonitis. There was no significant correlation between peritonitis rate and PHQ-9 score (r = − 0.001, *p* = 0.9) or frailty score (r = 0.042, *p* = 0.5). The correlation between PHQ-9 score or frailty score with peritonitis rate remains insignificant when only patients who performed self-PD were analyzed (PHQ-9 score: r = − 0.010, *p* = 0.880; frailty score: r = 0.075, *p* = 0.252).

### Patient survival

**Fig. 2:**
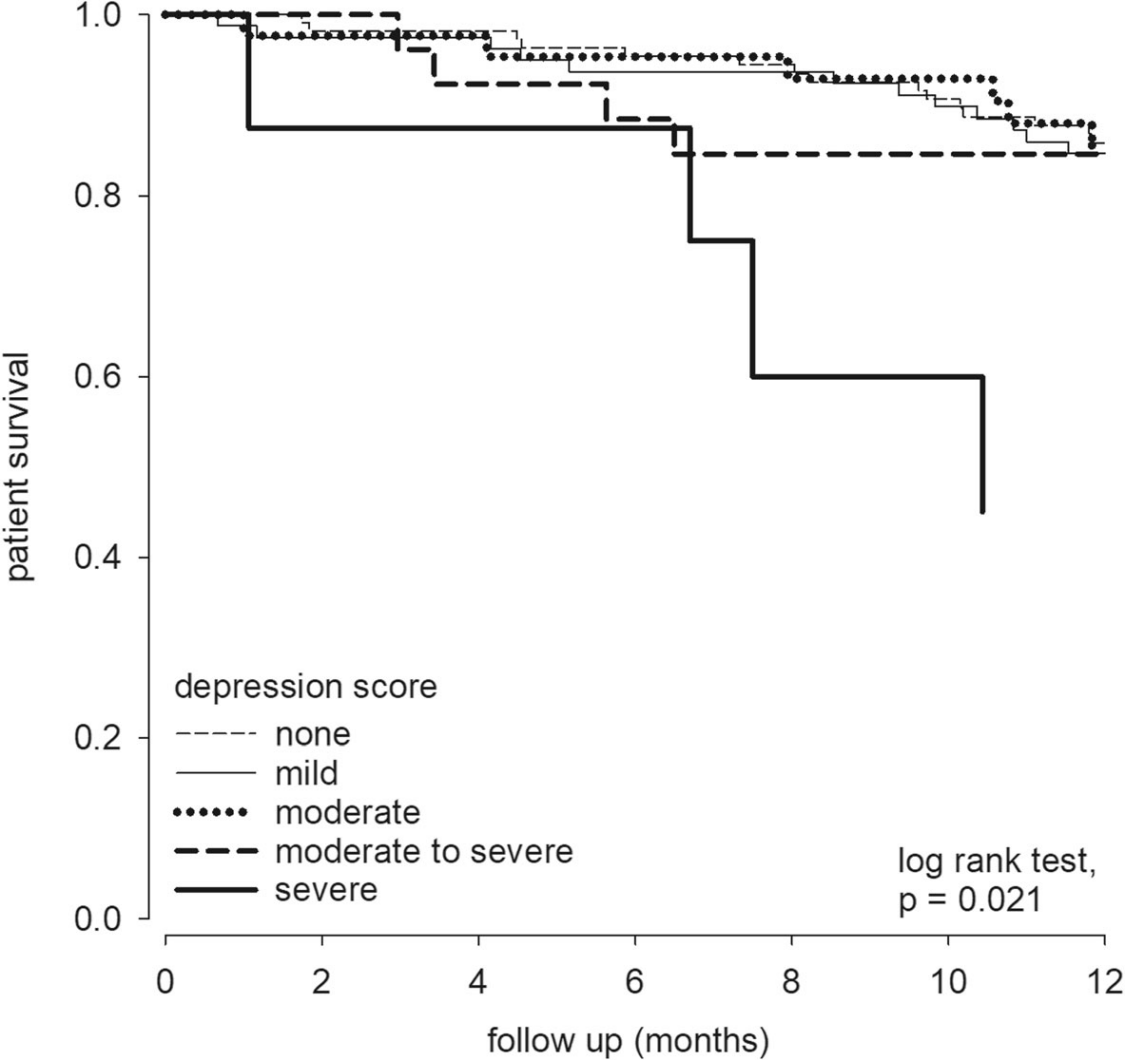
Kaplan-Meier plot of patient survival according to the severity of depression. We observed a significant difference between 1-year survival according to the severity of depression. Log-rank test, *p* = 0.021

**Fig. 3:**
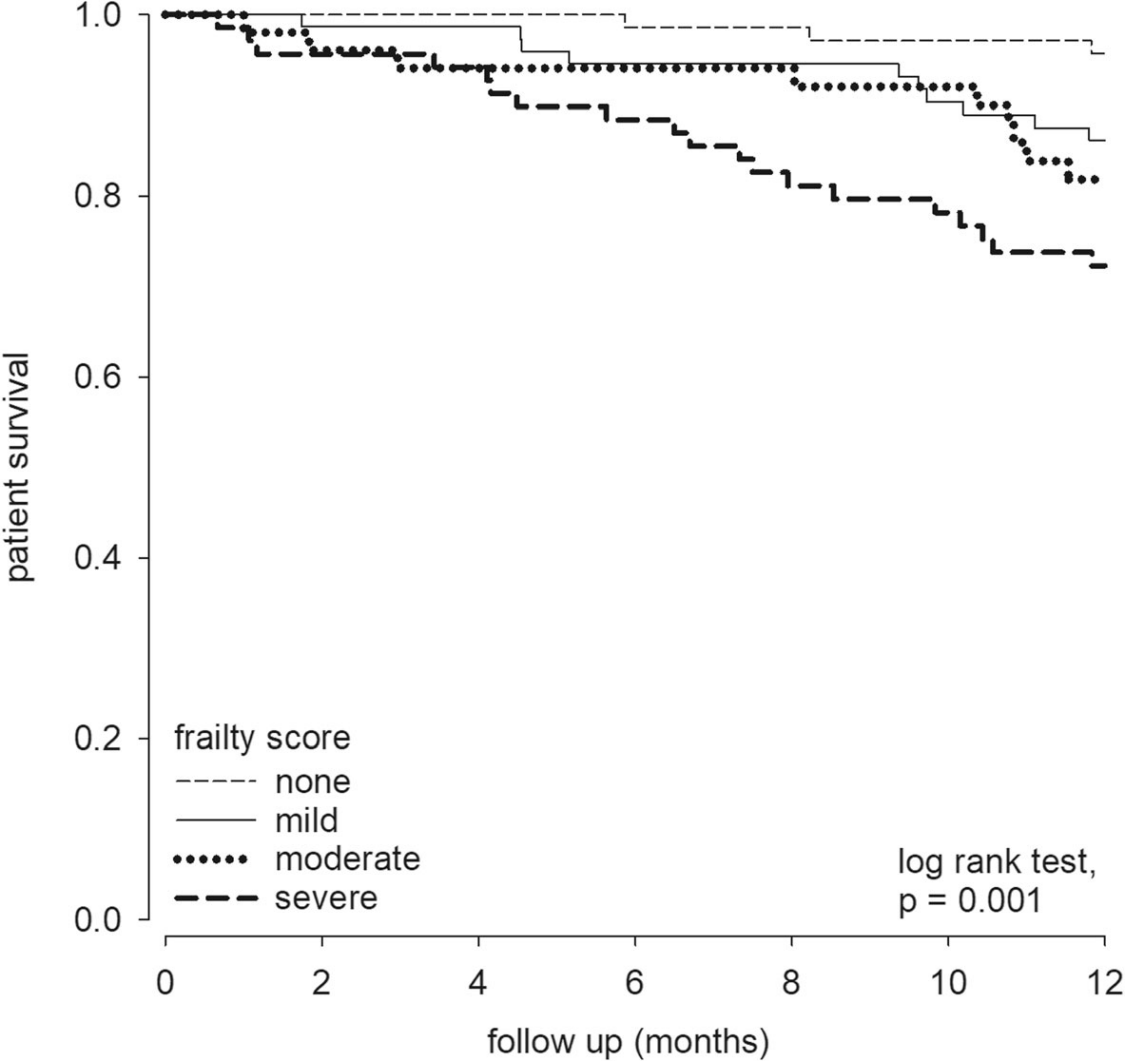
Kaplan-Meier plot of patient survival according to the severity of frailty. The 1-year survival rate significantly reduced according to the severity of frailty in a stepwise manner. Log-rank test, *p* = 0.001

During the study period, 43 patients (16.1%) died. During this period, 9 patients (3.4%) underwent kidney transplantation, 9 (3.4%) were switched to chronic hemodialysis, and 2 (0.7%) were transferred to other renal center. The overall one-year patient survival rate was 86.4, 85.0, 86.0, 80.8, and 50.0% for patients with nil, mild, moderate, moderate-to-severe, and severe depression, respectively (log rank test, *p* = 0.001) (Fig. 2). The corresponding one-year technique survival was 81.8, 82.5, 76.7 and 37.5%, respectively (log rank test, *p* = 0.046). As for frailty, one-year patient survival was 95.9, 86.5, 82.4 and 71.0% for patients with nil, mild, moderate and severe frailty, respectively (*p* = 0.001) (Fig. 3), and the corresponding one-year technique survival was 90.4, 81.1, 76.5, and 71.0%, respectively (*p* = 0.003). After adjusting for potential confounding factors by multivariate Cox regression analysis, frailty was an independent predictor of patient survival (adjusted hazard ratio [AHR] 1.424, 95% confidence interval [CI] 1.011–2.005, *p* = 0.043). In addition, Charlson Comorbidity Index and serum albumin level were independent predictors of patient survival (Table 4), but depression was not an independent predictor. There was also no interaction between PHQ-9 score and frailty score in predicting patient survival or technique survival.

**Table 4:** Multivariate Cox regression analysis of patient survival

Factors	AHR	95%CI	*P* value
Charlson comorbidity index	1.258	1.058–1.497	*p* = 0.010
albumin	0.863	0.797–0.934	*p* < 0.0001
frailty score	1.424	1.011–2.005	*p* = 0.043

*AHR* adjusted hazard ratio, *CI* confidence interval

## Discussion

Our study shows that depression and frailty are common in prevalent PD patients. Although neither depression nor frailty predicted hospitalization and peritonitis, we observed a significant relationship between mortality and frailty, but not with depression in PD patients.

Depression is the most common psychiatric problem in patients with end stage renal disease [20]. One of the most common diagnostic tools for depression is the Patient Health Questionnaire (PHQ-9) questionnaire, which was designed by Spitzer, Williams and Kronenke in 2001 [18]. PHQ-9 is based on the diagnostic criteria for Major Depressive Disorder under the code of Diagnostic and Statistical Manual of Mental Disorders, 4th Edition (DSM-IV). It is chosen due to many of its advantages. The use of this questionnaire is free of charge, and it is shorter than many other depression rating scales. PHQ-9 can be administered in person, over the phone or as self-report. It also facilitates the diagnosis of major and minor depression, and assesses the severity of depression symptom. It is available in multiple languages including traditional Chinese, which was used in our study. PHQ-9 questionnaire has also been validated in dialysis population [21]. PHQ-9 is sensitive and specific at identifying depression in general population [18] and in dialysis population [21]. However, it should be noted that PHQ-9 is best used as a monitoring tool for the assessment of depression severity rather than as a screening tool, and a slightly elevated PHQ-9 score (i.e. the “mild depression” group) may also always imply an actual diagnosis of depression. The use of PHQ-9 in this study may explain the high rate of depression in our study.

As for frailty, there are a few validated tools in measuring frailty. The Frailty Index (FI) is a tool to quantify and summarize patient’s vulnerability. It includes assessment of factors including of symptoms, underlying concomitant diseases, conditions and disability [22]. There are also other tools including Cardiovascular Health Study (CHS) scale [23], Study of Osteoporotic Fracture (SOF) scale [24], Canadian Study of Health and Aging (CSHA) Clinical Frailty Scale [25], to assess frailty. However, all of these scales require direct patient examination, which is time consuming and impractical as the screening test in large number population. Apart from technical difficulties, not all of the above-mentioned screening tools have been validated in CKD cohorts. In our study, we used a questionnaire developed by our university and has been validated in the Chinese population [6]. The questions are straightforward and easy to answer, which simplify the process of screening.

The prevalence of depression in our study was 58.8%. The result is comparable to another local study, which reported 55% prevalence of depression among Chinese PD patients [4]. On the other hand, the worldwide prevalence of depression among dialysis population is reported to be 20–30% [26]. However, most of the western studies focus on hemodialysis patients. A study in Korea [27] and in Hong Kong reported 75 and 43.2% [13] prevalence of depression respectively among the population undergoing CAPD. Such discrepancy could be explained by differences in in cohort size, characteristics of subjects, and the measurement tool for depression. We believe our result is more representative as the cohort size is much larger. We also adopted a more appropriate depression score to our middle-age dialysis patients.

At the first glance, it seems an unusually high figure to have over 70% of patients who required hospitalization within one year. It should be noted, however, that our local policy mandates hospital admission for many intervention procedures, such as cardiac catheterization and colonoscopy. Unfortunately, we do not have the detailed breakdown on the cause of hospitalization for our patients, but the figure in this study is similar to our previous reports [28, 29].

We did not find a significant association between depression and the number or duration of hospitalization after adjusting for clinical confounding factors. Although previous studies reported that depression in patients with end stage renal disease is significantly associated with adverse medical outcomes, including the number of hospitalization [30] and cumulative hospital days [31], these studies were based on hemodialysis patients and may not be applicable to our patients. A local study showed that depression is significantly associated with mortality rate in Chinese peritoneal dialysis patients [4]. However, this study only recruited patients who were newly started on PD.

In our study, depression was not an independent predictor of mortality after adjusting for frailty and other clinical confounding factors. In contrast, most of the previous studies reported an association between depression and mortality in dialysis patients. For example, Lopes et al. [32] noted an approximately 40% increase in mortality in patients who indicated the presence of depressive symptoms on self-reported questionnaires, although the study did not deploy any depression scale and the patients were not grouped according to their depression severity. A meta-analysis reported the mortality risk in patients on dialysis was 1.5 times higher in the presence of depressive symptoms after adjusting for other confounding factors [5]. However, none of the previous study on depression adjusts for frailty, even though the two parameters have a close internal correlation.

The prevalence of frailty in our study population was 72.7%, which is similar the prevalence of 73% reported by Bao et al. [33], and 67.7% by Johansen et al. [10]. In analyzing the baseline demographics and clinical parameters in our study population, we noted that frailty score had significant correlations with Charlson Comorbidity Index and dialysis adequacy. Correlation of frailty and comorbidities in dialysis patients was also reported in a prior study [34].

In our study, frailty score is better correlated with the number of hospitalization and duration of hospitalization than depression score. However, after adjusting for the confounding factors by multivariate analysis, frailty score was not a significant predictive factor of the number or duration of hospitalization. Our result is in contrary to prior studies. For example, Johansen et al. [10] showed that frail patients were more likely to be hospitalized for any reason even after adjusting for other potential risk factors for hospitalization [10]. McAdams-Demarco et al. [35] reported that frailty was a predictor of hospitalization in hemodialysis. Inclusion of hemodialysis patients in these studies may account for the discrepancy of their results with our study. Other possibilities include difference in patients’ ethnicity and age distribution.

With multivariate Cox regression analysis, our study showed that frailty was a significant predictor of all-cause mortality. Our result is in line with previous studies. For example, Alfaadhel et al. [36] demonstrated that frailty at the initiation of dialysis was associated with mortality. Johansen et al. [10] team showed that frailty was independently associated with the risk of death. We believe that frailty screening can help to identify a high risk group.

There are several limitations of our study. First, it is a single-center study of limited sample size, which affects the external validity and statistical power to detect subtle subgroup effects. Because of the limitation in our original study design and available data, we did not measure cognitive impairment, which is an integral part of frailty, and concurrent medications use e.g. antidepressants which may impact on depression. The follow up duration of one year is also not sufficient to detect any effect on patient mortality. Second, our study population was entirely Chinese. Because of the potential contribution of genetic and environmental factors to the mortality risk, our result may not be applicable to patients from other parts of world or even other parts of China. Our study only examined the rate of adverse outcome after one year, which may not be sufficient for mortality. Since our study recruited prevalent PD patients, the onset of depression and frailty (i.e. before or after dialysis was initiated) was not known. It was also possible that survivor bias was present, which may affect the result of our study.

## Conclusion

Our study shows that depression and frailty are common in prevalent PD patients. There is also a significant relationship between frailty and mortality in PD patients. Further studies are needed to determine the benefit of treatment for frailty in PD patients.

## Supplementary information

**Additional file 1.** Simplied frailty assessment questionnaire used in this study. A total score was calculated and the degree of frailty was divided into nil (score 5 or below), mild (score 6–8), moderate (score 9–11), or severe (score 12 or above).

**Additional file 2.** All raw data of our present study.

## Data Availability

All study data are listed in Supplementary File 2.
