# Supplementary figures and images for: Depression does not predict clinical outcome of Chinese peritoneal Dialysis patients after adjusting for the degree of frailty
Source: BMC Nephrol. 2020 Aug 5;21:329. doi: 10.1186/s12882-020-01994-4 (PMC7405374; doi:10.1186/s12882-020-01994-4)

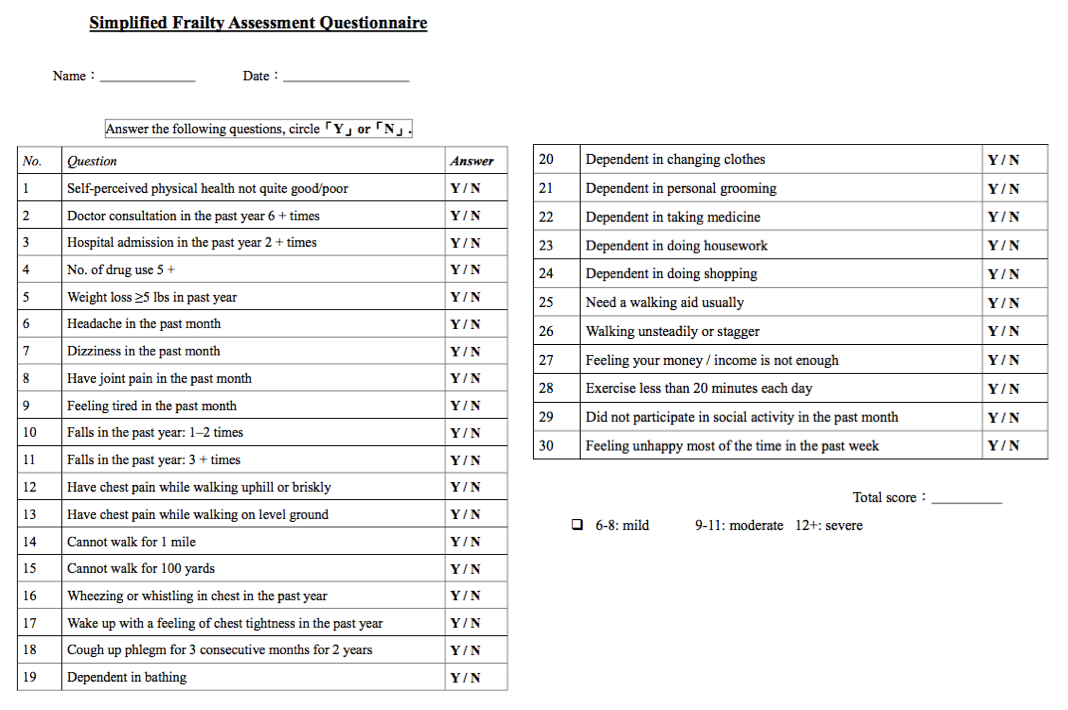

Supplement: Supplementary file 1 — Additional file 1. Simplied frailty assessment questionnaire used in this study. A total score was calculated and the degree of frailty was divided into nil (score 5 or below), mild (score 6–8), moderate (score 9–11), or severe (score 12 or above). [file 12882_2020_1994_MOESM1_ESM.tiff]
